# Analysis of gunshot injuries to the different surfaces of the shaft of a porcine femur with various lead airgun pellets fired from a 6.35 mm calibre air rifle using a human thigh model

**DOI:** 10.1371/journal.pone.0343400

**Published:** 2026-03-02

**Authors:** Mateusz Wilk, Elżbieta Chowaniec, Karol Jędrasiak, Piotr Wodarski

**Affiliations:** 1 Collegium Medicum, WSB University, Dąbrowa Górnicza, Poland; 2 GC Adwokaci Partnership, Katowice, Poland; 3 Department of Transport and Computer Science, WSB University, Dabrowa Gornicza, Poland; 4 Department of Biomechatronics, Faculty of Biomedical Engineering, Silesian University of Technology, Gliwice, Poland; University of Perugia: Universita degli Studi di Perugia, ITALY

## Abstract

Modern air weapons are approaching firearms with their capabilities. Shots from this type of weapon pose an increasing threat to humans and animals. The vast variation in airgun ammunition means that gunshot damage can vary depending on the type of bullet that hits the tissues. The study was undertaken to investigate how the type of lead airgun pellet calibre of 6.35 mm and surface of femoral shaft affected by gunshot damage have impact on extent of bone and periosteal entry damage. In our study, we subjected a porcine femur contained in a ballistic gelatine human thigh model to damage. 3 types of Haendler&Natterman (H&N) airgun pellets were fired at the anterior, posterior and lateral surfaces of the femoral shaft. The impact energies of the projectiles were measured. The properties of the femoral shaft were evaluated. The models were then subjected to X-ray imaging and slicing for photographic evaluation of pellet shot damage. The surface area of inlet damage in the bone and periosteum was measured, and the ratio of these dimensions to each other was calculated. It was noted that the posterior wall of the porcine femoral shaft is the thickest, and the lateral wall has the greatest variation in thickness. As a result of statistical analysis, it was noted that for the front surface of the femoral shaft, H&N Baracuda Hunter shot generated the biggest damage area and H&N Silverpoint the smallest; for the posterior surface, H&N Baracuda Hunter biggest and H&N Baracuda smallest damage area respectively; and for the lateral surface, H&N Silverpoint the biggest and H&N Baracuda the smallest damage area respectively. This is most likely influenced by the properties of the individual airgun pellets and the different thicknesses of the femoral shaft walls, including significant variation in the thickness of the lateral wall.

## Introduction

The type of damage caused by a gunshot depends on the characteristics of the bullet, the impact energy and the location of the hit. Ammunition for airguns available on the market varies in penetration properties, which affects the type of damage, depending on the use of the weapon for activities such as sport, competitive shooting or hunting. Over the past 20 years, airguns have undergone significant development, with modern rifles and pistols featuring higher energy discharges, bringing them closer to the ballistic performance of traditional firearms [1]. These changes have also affected ammunition, which now includes airgun pellets of different calibres, shapes and materials. Given the ease of modification of commercially available air rifles, research has begun on femur damage caused by shots fired from 6.35 mm calibre air rifles. To date, few studies have focused on bone damage caused by high-powered air rifles. In the past, airgun-induced femur injuries have been studied using 0.25-inch (6.35 mm) and 0.406-inch (10.03 mm) steel pellets fired at immobilised human femurs not encased in ballistic gelatine [2].

Authors found that found that the larger projectiles expended significantly more energy in fracturing a femur than the smaller projectiles did at an identical impact velocity. Moreover, the larger spheres expended more energy in fracturing the femur and impacts to cortical bone of the femoral shaft by either size projectile caused treater energv expenditure than impacts to the distal end of the femur.

There is a paucity of information in the literature on high-energy airgun injuries, generaly studies of firearm-related bone damage and internal organ injuries caused by low-energy pneumatic devices are better described [3]. For example, the behaviour of the femur in contact with a bullet has been studied, but without considering the effect of soft tissues. In 2010, experiments were conducted on long bone damage from a 15.3 J airgun pellet by shooting bovine femur fragments surrounded with ballistic gelatine [4] It was found that even with this impact energy, no damage could have been observed on the bone and most of the CT scans appeared to show no evidence of density change in the bone due to compaction, although some CT scans may suggest some possibility of local damage. As a result authors suggested that model their proposed need further investigation and development.

In 2018, a study was published on the penetration of a porcine femur by a fragment simulating a projectile (FSP) that passed through a single layer of bone without damaging the entire shaft [5]. In this study, preliminary experiment was conducted with porcine femur surrounded with a block of 20% 300 Bloom ballistic gelatine as a target. A 150 N compression, was applied longitudinally to the sample to simulate the boundary condition of the standing posture. The 0.78 g cylindrical carbon-steel FSP was used with impact velocity of 326 ± 5 m/s. Calculated impact energy was around 41.5J. Only one shot was carried out with resulting puncture in one face of femur shaft but unfortunately without information about dimensions of periosteal and bone damage.

Shortly thereafter, a study showing a correlation between FSP energy and damage to the fibula of a sheep was published to simulate damage to the fibula of a 5-year-old child [6]. In this case, the bones were not surrounded with ballistic gelatine, making it impossible to assess damage to bones surrounded by soft tissue. Authors however used modified Winquist-Hansen fracture classification applied to the tibia impacted by a small metallic fragment-simulating projectile. Single shots at gradually increased impact energy were performed. Authors calculated probability of fractures for 3 different fibula faces, but did not describe dimensions of shot damage to the bone.

Another study looked at the damage caused by shots fired with 4.5 mm calibre pellets from a CO2 gun, where four human feet were shot, which were later X-rayed and dissected [7]. The impact Energy was calculated as around 4 J. The results of this experiment were presented in the form of photos and tables. Authors found, that a high penetrative capacity of all the used pellets was observed with exception of the wadcutter type of pellet. Penetrations in soft tissues were associated with bone lesions, such as abrasions of the cortical bone, chipping and/or fractures, which in some cases were even comminuted (however multiple soths were performed for single human foot and mostly cancellous bone was targeted). Microscopically, metallic traces, bone spicules, and striae-like irregularities on the bottom of all the bone abrasions were found. Unfortunately, no dimensions of bone damage were produced.

In 2025 our team performed another study trying to find a new model and new method of shot damage description [8]. The study used the same model as in the current study. A 5.5 mm calibre airgun was used with four types of pellets, including lead-free pellets. Impact energy ranged between 27 and 44 J depending on airgun pellet type. After the shooting, CT scans were performed and a visual assessment of bone and periosteal damage was made. It was found that hollow-point pellets caused the most damage to the periosteum and bone, while lead-free pellets caused the least damage due to their penetrating nature. Due to the omission of areas of the pig femur other than the anterior surface in that study, it was decided to expand the project with another study based on the assessment of post-shot damage to the femur using a larger calibre 6.35 mm pneumatic weapon, which generates greater impact energy.

## Study objectives

The study was undertaken to investigate how the type of lead airgun pellet calibre of 6.35 mm and surface of femoral shaft affected by gunshot damage have impact on extent of bone and periosteal entry damage.

## Materials and methods

Before beginning of the studies, approval from Local Ethical Committee for Animal Experiments in Katowice, Poland, was obtained (issue PCN/022/LKE/4/20).

The first stage of the research involved calibration of ballistic gelatine, for which the Crosman C2100 airgun and Razorgun pellet calibre 4.46 mm were used. Pellet impact velocities of approximately 162–167 m/s were obtained, yielding average penetration results in gelatine ranging from 72–80 mm according to the procedure [9]. All stages of the research were performed between February and March 2025 to ensure stable and low but above 0^o^C temperature.

The study used 3 types of Haendler&Natterman (H&N) 6.35 mm calibre (cal.) airgun pellets: Baracuda, Baracuda Hunter, Silverpoint.

**Fig 1:**
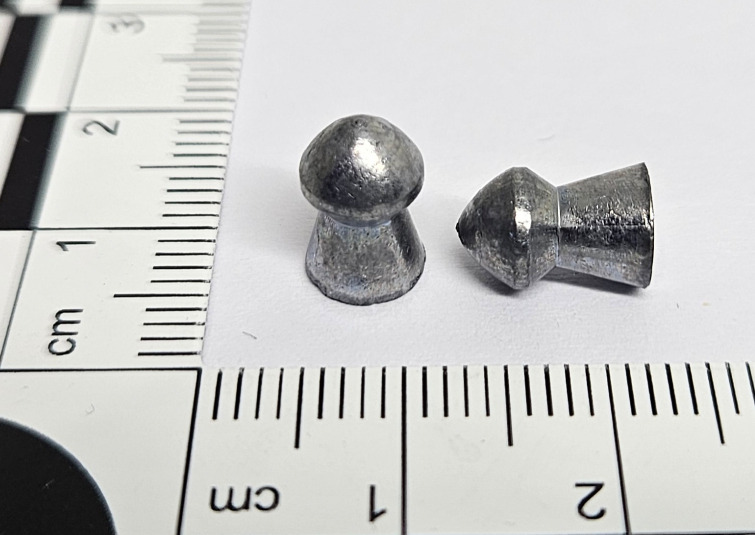
H&N Baracuda airgun pellet cal. 6.35 mm.

The H&N Baracuda airgun pellet (Fig 1) is a heavy pellet with a slightly rounded conical tip part (head). Its shape is a combination of features of pointed and mushroom-shaped pellet, however, it is slimmer (longer at the same calibre). At the same time, it is much heavier than typical diabolo type pellets of the same calibre. Due to its weight, it is used in the most powerful air rifles. The weight of the pellet is 2.01g [10].

**Fig 2:**
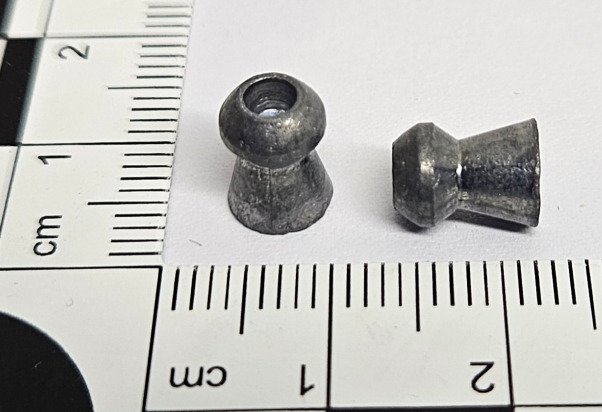
H&N Baracuda Hunter airgun pellet cal. 6.35 mm.

H&N Baracuda Hunter airgun pellet (Fig 2) is a pellet with a characteristic indentation of the central part of the head, resembling burst-type ammunition giving it fragmentation propereties. Such a construction of the pellet, after hitting the target, causes deformation of it leading to a significant degree of flattening) and fragmentation. The weight of the pellet is 1.78g [11].

**Fig 3:**
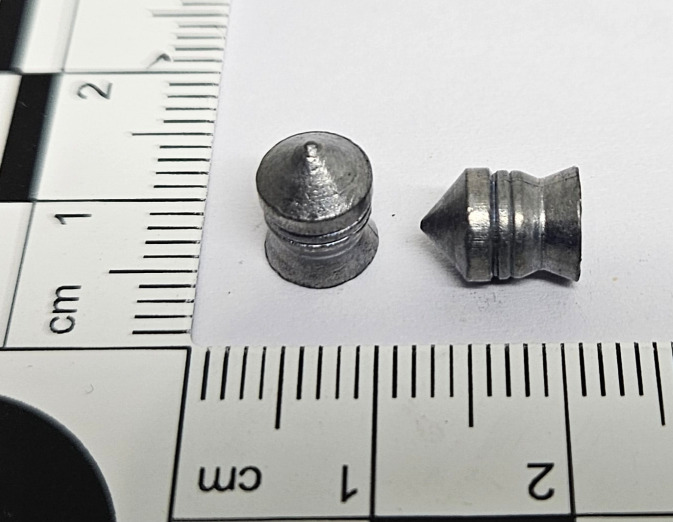
H&N Silverpont airgun pellet cal. 6.35 mm.

H&N Silverpoint (Fig 3) is a sharp-pointed aigun pellet of moderate weight. The shape of its head makes it a pellet with good penetration properties, at the same time it reduces the risk of ricochet when hitting a soft obstacle. Conical sharp pointed head is followed by barrel-like pellet body, which increases weight of the pellet. It is a pellet characterised by good grouping at short and medium ranges. The weight of the pellet is 1.58 g [12].

**Fig 4:**
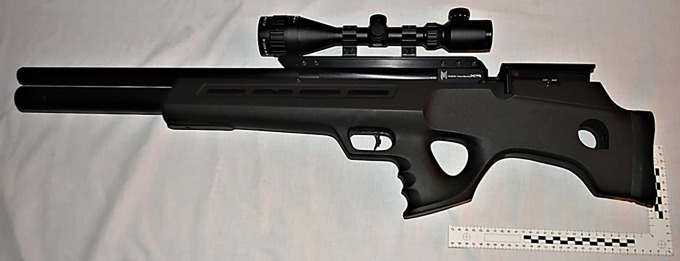
PCP FAC FX Bobcat Mk II cal. 6.35 mm air rifle.

A PCP FAC FX Bobcat Mk II cal. 6.35 mm (Fig 4) was used for shooting conducted at an indoor range. Depending on the weight of the pellet used, the maximum impact energy is about 65J [13].

The velocity and energy of the selected airgun pellets were measured using an LMBR R2A shooting chronometer [14]. The tests were conducted at a distance of 10m using the maximum power of the air rifle. Each measurement was repeated 10 times in order to obtain an optimal control sample and authoritative, reliable and methodologically correct results. Taking into account the data obtained, the kinetic energy of the airgun pellets at a distance of 10m was calculated, which at the same time was the impact energy of the airgun pellets on the human thigh model. The obtained energy results were verified with the results obtained with the LMBR R2A shooting chronograph, which had calculation functions for the parameters of the fired airgun pellets. Results of this stage of the study are shown in Table 1 and Table 2. Air rifle shots were fired perpendicularly to a specific surface of the femur shaft (anterior, posterior lateral), into the central area of the shaft surface, using a gun stabilising stand. The shooting chronograph was set at a distance of 10m from the barrel exit of the carbine. During the shooting, the pressure in the air rifle cartridges was constantly monitored. Ten shots were fired at each surface of the shaft with a given type of pellet.

**Table 1:** Velocities of cal. 6.35mm airgun pellets measures at a distance of 10m, [m/s].

Name of pellet	Mean	SD	Max	Min
H&N Baracuda	242.3	1.11	244.8	240.2
H&N Baracuda Hunter	257.2	0.72	262.3	250.2
H&N Silverpoint	270.2	0.92	271.1	260.7

**Table 2:** Impact energy values of cal. 6.35 mm airgun pellets measured at a distance of 10m, [J].

Name of pellet	Mean	SD	Max	Min
H&N Baracuda	59.0	1.11	60.2	57.9
H&N Baracuda Hunter	58.8	0.72	61.2	55.7
H&N Silverpoint	57.6	0.92	58.0	53.6

Fresh porcine femur bones were mechanically cleaned of soft tissues. The bones came from a slaughter performed on the same day in the morning hours. The weight of the pigs from which the femurs were obtained was about 120 kg. The parameters of the porcine femurs used in the study are listed in the Table 3.

**Table 3:** Parameters of porcine bones used in the study (A-P: front – back, L-L: side – side, SD: standard deviation, Max: maximum value, Min: minimum value).

Parameter	Mean	SD	Max	Min
Overall length [mm]	244.25	5.85	267.10	222.15
Diameter of the bone shaft A-P [mm]	29.56	1.65	35.69	26.39
Diameter of the bone shaft L-L [mm]	29.22	1.86	32.45	27.50
Average cortical thickness of the anterior shaft of the bone [mm]	4.22	0.22	4.49	4.19
Average cortical thickness of the posterior shaft of the bone [mm]	4.44	0.29	4.92	4.62
Average cortical thickness of the lateral shaft of the bone [mm]	3.92	0.88	5.55	3.25
Weight of bone [g]	410.21	19.96	460.00	375.00

According to the literature, despite a similar external diameter measured at half its length, the dimensions of the human femoral shaft differ slightly from the external dimensions of the porcine femoral shaft used in the study (human shaft of femur being slightly narrower than porcine femur – 26–28 mm) [15–17]. Significant differences occur in the thickness of the cortical layer of the human bone shaft compared to the parameters used in the study of porcine femurs. Average cortical thickness of femur shaft in central part was around 5.5 mm, with posterior and medial surfacer reaching up to 6 mm and the thinnest being anterior and lateral surfaces [16]. In another study describing properties of human femur in the middle of the shaft, femur shaft medial cortical thickness was 6.8 mm, and lateral cortical thickness was 6.9 mm [17].

The human thigh model was a porcine femur embedded in a cylinder of 10% ballistic gelatine cooled to 4 degrees Celsius, with an outer diameter of about 155 mm and a height of about 250 mm prepared according to the procedure [9,18]. The femur was positioned vertically and centrally in the ballistic gelatine cylinder so that the distance from the walls of the thigh model was the same for each of the walls tested.

10% solution of 240 Bloom pork gelatine was prepared to fill the casting tubes with bones inside. The FBI model (10% concentration, gelatine at temperature 4°C) [18] was chosen due to the better transparency of the ballistic gel compared to the US Army model (20% concentration, gelatine at temperature 10°C). This was crucial for the study, as good gelatine transparency was an important factor enabling macroscopic assessment of post-shot damage and imaging using visible light (photographs). During the preparation of the 10% gelatine solution, it was important to maintain a temperature not exceeding 60–65°C and to stir continuously until the gelatine crystals dissolved in the solution. After preparing the solution, a small amount was poured into a plastic container measuring 20 cm x 20 cm x 5 cm in order to perform a gelatine calibration test after cooling the sample to 4°C. Calibration using 4.46 mm steel balls and a Crosman C2100 4.5 mm rifle was performed according to the standard [8] at an indoor shooting range. The calibration used pellet impact velocities of approx. 162–165 m/s, obtaining average penetration results in gelatine in the range of 72–78 mm.

The time between obtaining the bones by cutting up pig carcasses and making the thigh models did not exceed 3 hours, after which the filled with 1 femur bone and previously prepared ballistic gelatine moulds were cooled at 4 degrees Celsius for 10 hours. Then, shooting tests were carried out in an indoor shooting range. The tests were conducted under constant and unchanging conditions of temperature (+3°C to +5°C), air humidity of 60%, artificial lighting and windless conditions, which was necessary to ensure the repeatability of the results obtained by eliminating the influence of varying atmospheric and environmental conditions, taking into account the ballistic parameters of the pellets fired and the risk of destabilising the flight path of the projectiles. After the shootings, the models were subjected to X-ray imaging in A-P and lateral projection and sectioned to assess the gunshot damage using photographic documentation. Visual assessment of bone and periosteal damage was performed using digital magnification and then the following parameters were measured manually using ImageJ software (separately for each shot, calibre and surface area of bone entrance): Surface area of the bone entrance wound and Surface area of the periosteum entrance wound. The ratio of the surface area of periosteum inlet to the surface area of bone inlet (RA ratio) was also calculated, which was justified by the observed differences in the shapes and sizes of gunshot damage dependent on the different types of airgun pellets used.

As part of the statistical analysis of the research results, positional parameter estimators, expected values and standard deviations were determined for all variables; in addition, the hypothesis of normal distribution was verified using the Shapiro-Wilk test.

The verification of statistical hypotheses concerning the comparison of the analysed samples, given the positive verification of the normality of the distribution in each case, was carried out using parametric tests: a test for two means preceded by a test for two variances, a test of homogeneity for multiple means preceded by a test of homogeneity of multiple variances (Bartlett's test and Levene's test). Depending on the results of the tests of homogeneity of multiple variances, the following were used:

a. the classic variant (one-way ANOVA) for cases of positively verified homogeneity of variance,
b. the Brown-Forsythe corrected variant for cases of negatively verified homogeneity of variance.

For those cases in which the result of the classical variant of the analysis of variance indicated significant differences, post-hoc tests in Fisher's LSD variant were used. In the opposite cases, Games-Howell post-hoc tests were used.

The results of the statistical analysis are presented in graphical form. The following levels of statistical significance were taken into account:

p > 0.05 – no statistical significance,
p < 0.05 – statistical significance,
p < 0.01 – high statistical significance,
p < 0.001 – very high statistical significance.

## Results

Legend for the article is shown in Table 4.

**Table 4:** Coding of different airgun pellets depending on surface of femur shaft shot.

First letter	Second letter	Number
A – anterior surface P – posterior surface L – lateral surface	B – H&N Baracuda H – H&N Baracuda Hunter S – H&N Silverpoint	6 – cal. 6.35 mm

In the following tables, the data (penetrations) are presented in the form of an X/Y system, where X denotes the number of bone surfaces penetrated and Y denotes the total number of shots fired, including ricochets. In the case of fractures of the femoral shaft, only cases where the bone wall was penetrated were taken into account, excluding ricochets. On the charts, the coloured dots surrounding the average result represent individual measurement results and their distribution in relation to other statistical parameters. In charts, “Bone inlet area” is a synonym to “Bone entrance wound area”, and “Periosteum inlet area” is a synonym to “Periosteum entrance wound area”

### Comparison of damage to the anterior surfaces of the femoral shaft

**Table 5:** Extent of bone damage depending on the type of 6.35 mm airgun pellets when shooting into to the anterior surface of the femoral shaft. Cases of ricochets were excluded from further analysis.

Type of pellet	Penetration of anterior surface bone	Ricochet	Fractures of anterior surface bone	Fractures of lateral surface bone	Fractures of posterior surface bone	Penetration of posterior surface bone
AB6	10/10	0	10/10	10/10	10/10	10/10
AH6	10/15	5	10/10	10/10	10/10	0/10
AS6	10/10	0	10/10	10/10	10/10	0/10

**Fig 5:**
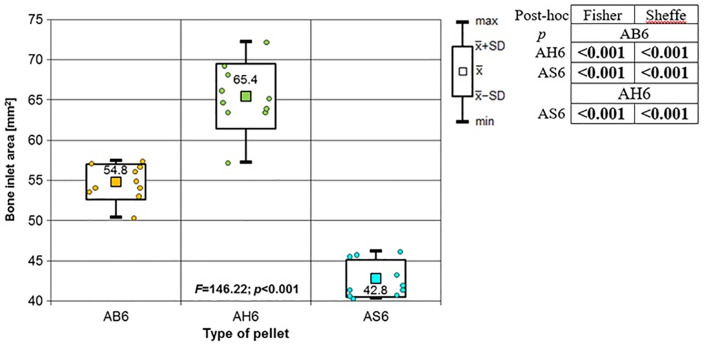
Bone entrance wound area in [mm^2^] depending to type of pellet (result of one way Anova and results of post-hoc tests: LSD Fisher and Sheffe). Shot to the anterior surface of the femoral shaft.

**Fig 6:**
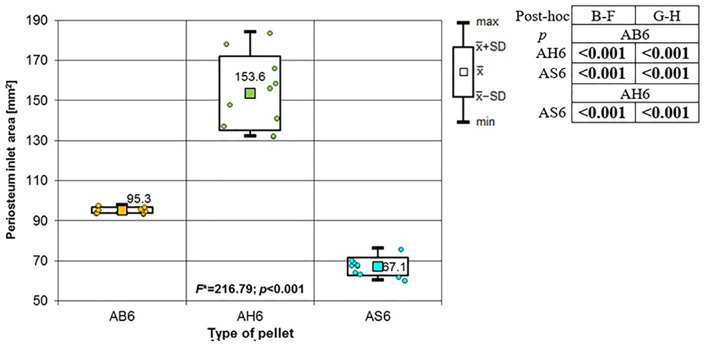
Periosteum entrance wound area in [mm^2^] according to the type of pellet (result of one way Anova with Brown-Forsythe correction and results of post-hoc tests: Brown-Forsythe and Games-Howell). Shot to the anterior surface of the femoral shaft.

**Fig 7:**
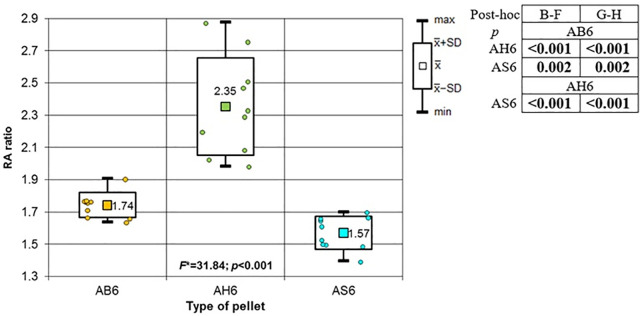
RA ratio depending on type of pellet (result of one way Anova with Brown-Forsythe correction and results of post-hoc tests: Brown-Forsythe and Games-Howell). Shot to the anterior surface of the femoral shaft.

### Graphic results

**Fig 8:**
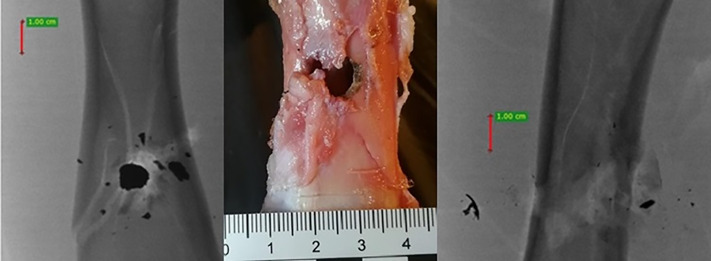
H&N Baracuda airgun pellet cal. 6.35 mm, on the left: A-P X-ray, centre: entry bone damage, on the right: lateral X-ray. Shot to the anterior surface of the femoral shaft.

**Fig 9:**
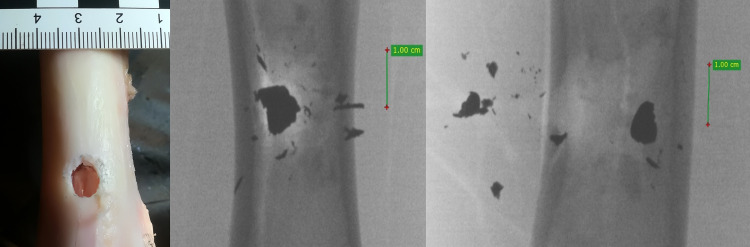
H&N Baracuda Hunter airgun pellet cal. 6.35 mm, from the left: entry wound damage, A-P X-ray, lateral X-ray. Shot to the anterior surface of the femoral shaft.

**Fig 10:**
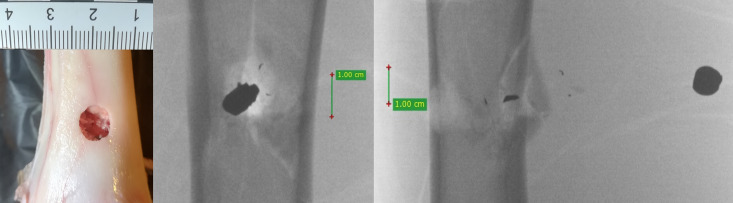
H&N Silverpoint airgun pellet cal. 6.35 mm, from the left: entry wound damage, A-P X-ray, lateral X-ray. Shot to the anterior surface of the femoral shaft.

### Comparison of damage to the posterior surfaces of the femoral shaft

**Table 6:** Extent of bone damage depending on the type of 6.35 mm airgun pellets when shooting into to the posterior surface of the femoral shaft. Cases of ricochets were excluded from further analysis.

Type of pellet	Penetration of posterior surface bone	Ricochet	Fractures of posterior surface bone	Fractures of lateral surface bone	Fractures of anterior surface bone	Penetration of anterior surface bone
PB6	10/10	0	10/10	10/10	10/10	0/10
PH6	10/15	5	10/10	10/10	10/10	0/10
PS6	10/12	2	10/10	10/10	10/10	0/10

**Fig 11:**
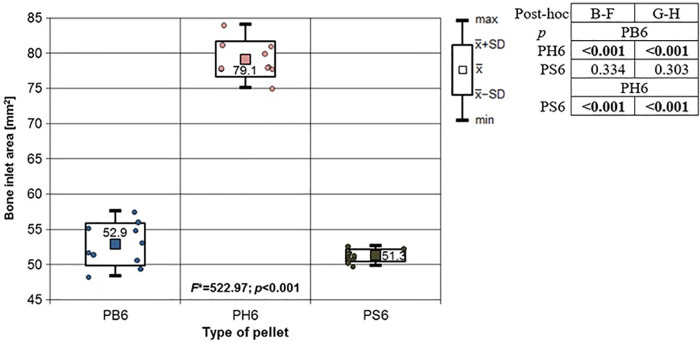
Bone entrance wound area in [mm^2^] depending to the type of pellet (result of one way Anova with Brown-Forsythe correction and results of post-hoc tests: Brown-Forsythe and Games-Howell). Shot to the posterior surface of the femoral shaft.

**Fig 12:**
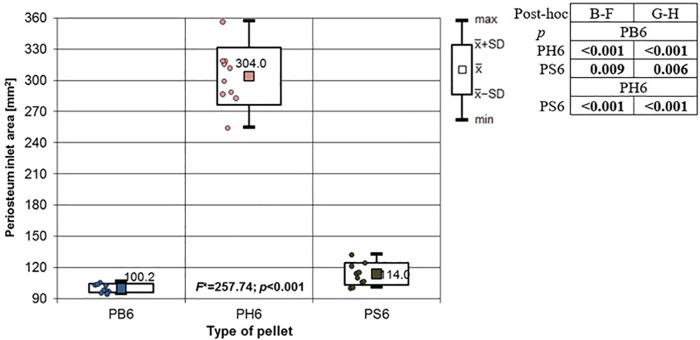
Periosteum entrance wound area in [mm^2^] depending on the type of pellet (result of one way Anova with Brown-Forsythe correction and results of post-hoc tests: Brown-Forsythe and Games-Howell). Shot to the posterior surface of the femoral shaft.

**Fig 13:**
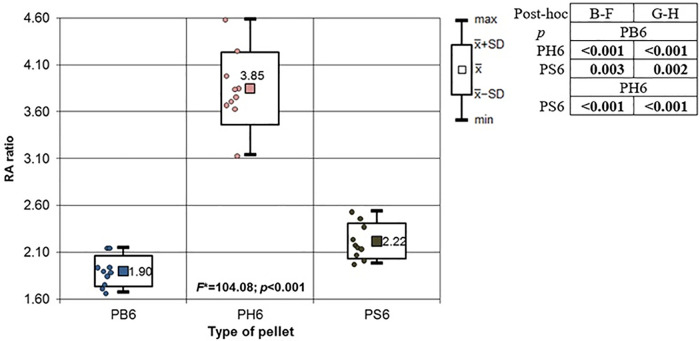
RA ratio depending on type of pellet (result of one way Anova with Brown-Forsythe correction and results of post-hoc tests: Brown-Forsythe and Games-Howell). Shot to the posterior surface of the femoral shaft.

**Fig 14:**
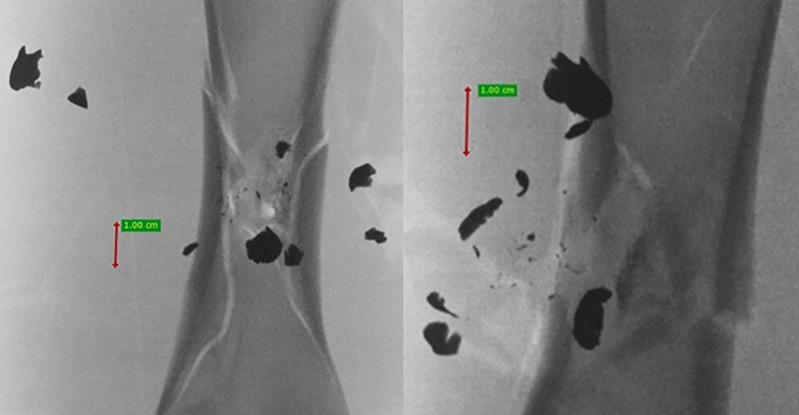
H&N Baracuda airgun pellet cal. 6.35 mm, on the left: A-P X-ray, on the right: lateral X-ray. Shot to the posterior surface of the femoral shaft.

**Fig 15:**
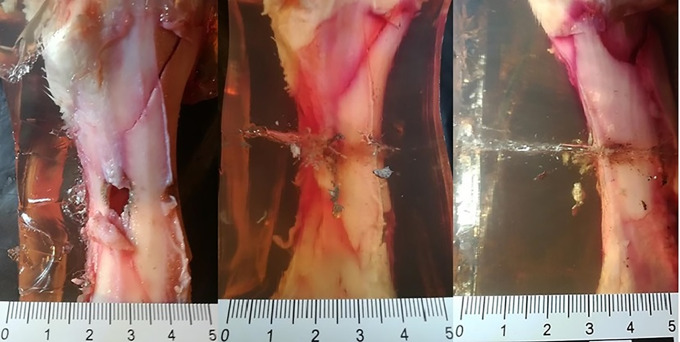
H&N Baracuda airgun pellet cal. 6.35 mm, on the left: entry damage, in the centre: view of shrapnel in front of bone, on the right: view of shrapnel in front of bone and detached periosteum. Shot to the posterior surface of the femoral shaft.

**Fig 16:**
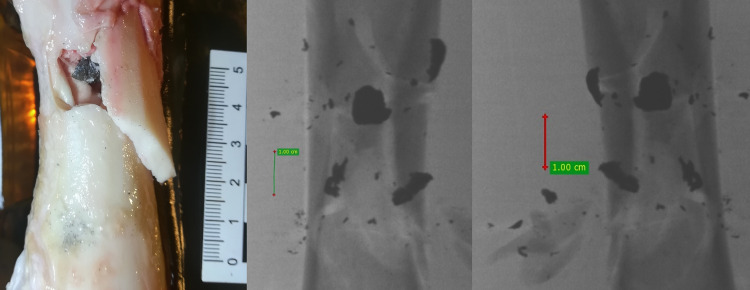
H&N Baracuda Hunter pellet cal 6.35 mm, from the left: entry wound damage, A-P X-ray, lateral X-ray. Shot to the posterior surface of the femoral shaft.

**Fig 17:**
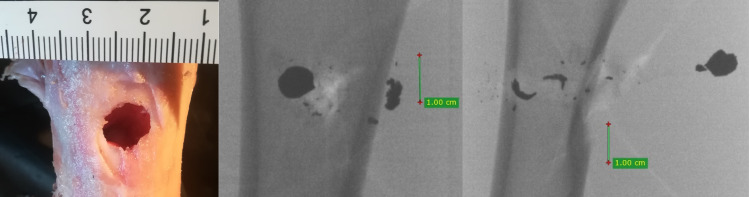
H&N Silverpoint airgun pellet cal 6.35 mm, from the left: entry wound damage, A-P X-ray, lateral X-ray. Shot to the posterior surface of the femoral shaft.

### Comparison of damage to the lateral surfaces of the femoral shaft

**Fig 18:**
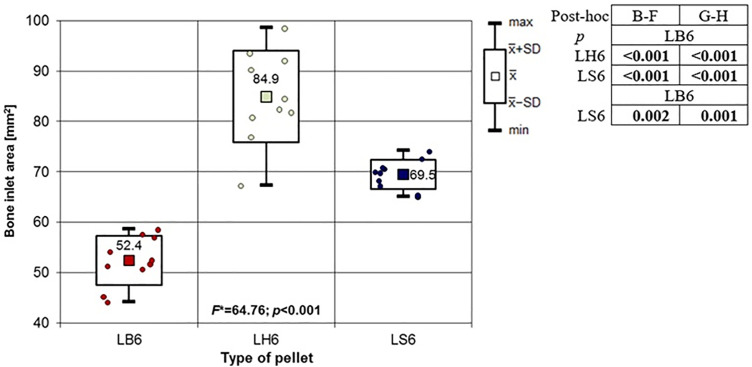
Bone entrance wound area in [mm^2^] depending to the type of pellet (result of one way Anova with Brown-Forsythe correction and results of post-hoc tests: Brown-Forsythe and Games-Howell). Shot to the lateral surface of the femoral shaft.

**Fig 19:**
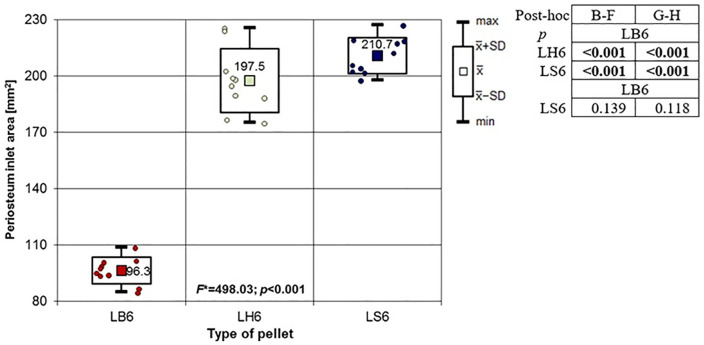
Periosteum entrance wound area in [mm^2^] depending on the type of pellet (result of one way Anova with Brown-Forsythe correction and results of post-hoc tests: Brown-Forsythe and Games-Howell). Shot to the lateral surface of the femoral shaft.

**Fig 20:**
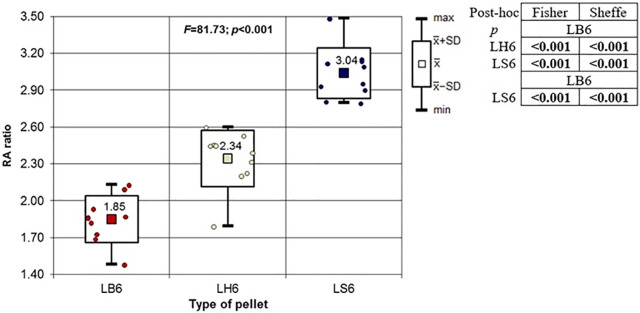
RA ratio depending on the type of pellet (result of one way Anova and results of post-hoc tests: LSD Fisher and Sheffe). Shot to the lateral surface of the femoral shaft.

**Fig 21:**
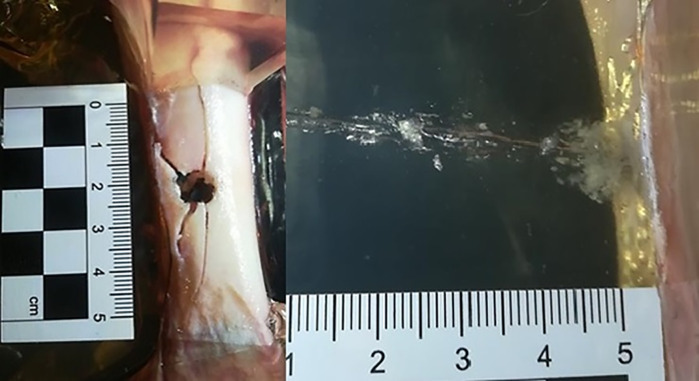
H&N Baracuda airgun pellet cal. 6.35 mm, on the left: image of entry damage, on the right: view of pellet fragments in front of bone and detached periosteum. Shot to the lateral surface of the femoral shaft.

**Fig 22:**
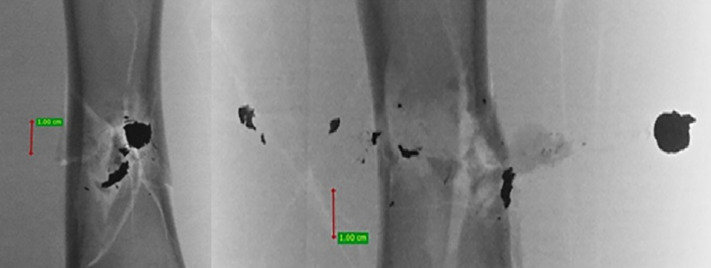
H&N Baracuda airgun pellet cal. 6.35 mm, on the left: A-P X-ray, on the right: lateral X-ray. Shot to the lateral surface of the femoral shaft.

**Fig 23:**
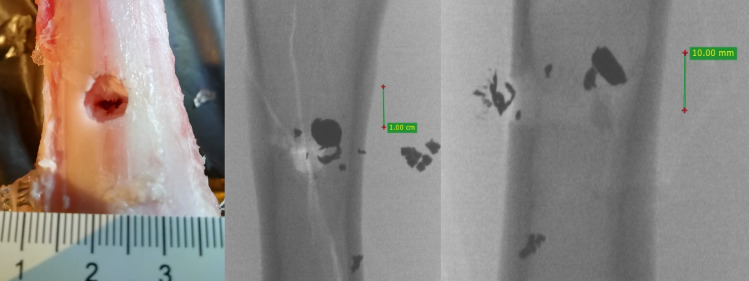
H&N Baracuda Hunter airgun pellet cal. 6.35 mm, on the left:A-P X-ray, in the centre: image of entry damage, on the right: lateral X-ray. Shot to the lateral surface of the femoral shaft.

**Fig 24:**
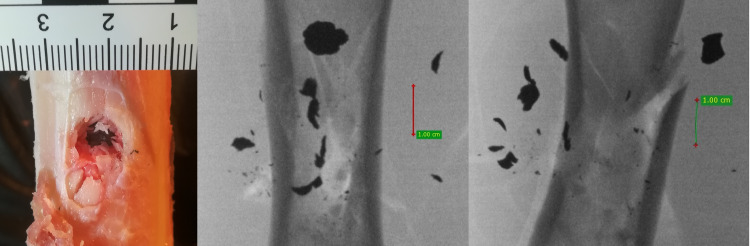
H&N Silverpoint airgun pellet cal. 6.35 mm, on the left: A-P X-ray, in the centre: image of entry damage, on the right: lateral X-ray. Shot to the lateral surface of the femoral shaft.

**Table 7:** Extent of bone damage depending on the type of 6.35 mm airgun pellets when shooting into to the lateral surface of the femoral shaft. Cases of ricochets were excluded from further analysis.

Type of pellet	Penetration of lateral surface bone	Ricochet	Fractures of lateral surface bone	Fractures of anterior/posterior surface bone	Fractures of medial surface bone	Penetration of medial surface bone
LB6	10/10	0	10/10	10/10	10/10	10/10
LH6	10/14	4	10/10	10/10	10/10	0/10
LS6	10/12	2	10/10	10/10	10/10	0/10

## Discussion

### Gunshot damage to the anterior surface of the shaft of porcine femur

Depending on the type, the 6.35 mm calibre airgun pellet caused gunshot damage to the bone and periosteum of different sizes, morphology and nature. Striking the bone, the bullet breaks off the oval entry hole, with the bigger dimension in the long axis of the shaft, which is surrounded by irregularly torn periosteum. The greatest damage within the entry holes was caused by shots with H&N Baracuda Hunter airgun pellets (Fig 5). A similar situation was observed in the case of damage to the periosteum (Fig 6). Also, the RA coefficient (the ratio of the area of periosteal entry damage to the area of the entry damage in the bone) shows the highest values for this pellet, which is determined by the characteristics of the pellet design itself (Fig 7). The shape of the H&N Baracuda Hunter pellet, in particular its flattened head with a central depression at the top, causes this airgun pellet to deform more when hitting a hard obstacle than the other pellets used in the study. After hitting the target, a pellet of this shape loses speed very quickly and deforms. Its deformed head creates a larger hole in the bone than is observed with the other pellets, while the flattened cup, together with the shock wave generated on impact, damages the periosteum over a much larger area than the other pellets. The direction of the fracture fissures is most likely due to the architecture of the femoral shaft, with osteon systems running in the long axis of the shaft, which determines the stress pattern in the shaft discovered by Koch [19].

The H&N Baracuda pellet, the heaviest of the 6.35 mm calibre pellets, was the only one to penetrate the shaft of the bone through. At the same time, shot fractures occur on both the front surface of the bone and the lateral and posterior surfaces (Table 5). This is most likely due to the significant mass and slower reduction of impact energy after contact with the target.

The entry holes in the shafts of the bones after being shot with 6.35 mm calibre pellets are oval in shape, very close to round. They are surrounded by an area of damaged periosteum, which is torn in an irregular manner (Fig 8). Analyzing the areas of damage to the periosteal entrance damage, it should be noted that, as in the case of the bone entrance damage, the greatest damage is caused by H&N Baracuda Hunter, while the least damage is caused by H&N Silverpoint. Analyzing the RA coefficient for individual 6.35 mm calibre pellets, it can be seen that it reaches the highest value for the H&N Baracuda Hunter pellet, and the lowest for the H&N Silverpoint pellet. The reason for such characteristics of the damage morphology caused by 6.35 mm calibre airgun pellets is that greatest extent of damage is caused by airgun pellets with a large surface area of the head, which offers greater resistance after hitting a hard obstacle and undergo significant deformation and even fragmentation. The least damage is encountered in shots with hard, deformation-resistant airgun pellets with a rounded or pointed head (Fig 9, Fig 10).

### Gunshot damage to the posterior surface of the shaft of the porcine femur

At the begining, it should be clarified that the bony layer of the posterior surface of the porcine femur is thicker than its anterior surface by about 13% - which needs to be taken into account when considering gunshot damage. This may be due to the fact that in the pig, as a four-legged animal, there is an increased pressure exerted by body weight on the posterior surface of the femoral shaft.

The 6.35 mm calibre airgun pellets ricocheted when shot at the posterior surface of the porcine femur, with the highest number of ricochets involving the H&N Baracuda Hunter pellet (Table 6), which is due to the shape of its head, which is flat with an indentation at the tip. Of the pellets, all caused fractures in the bone on both the front, rear and lateral surfaces. All fracture fissures were similar in nature to the gunshot injuries described of the anterior surface of the bone shaft however, they were more extensive than them (Fig 11, Fig 12, Fig 13). In the case of H&N Baracuda and H&N Baracuda Hunter, fractures of the bony walls of the shaft were more numerous (Fig 14, Fig 15, Fig 16).

The airgun pellet that caused the most damage to the bone after being hit was, as in the case of previously described gunshots, H&N Baracuda Hunter, while the least damage was caused by H&N Baracuda and H&N Silverpoint pellets. Damage to the periosteum caused by hits with 6.35 mm calibre airgun pellets is similar in nature to that described in gunshots to the front surface of the bone with the greatest damage caused by hits with H&N Baracuda Hunter pellets, while the least damage was produced by H&N Silverpoint pellets (Fig 17).

Analyzing all pellets fired into the anterior and posterior surfaces of the bone, of particular note is the RA coefficient, which is the ratio of the area of periosteal entry damage to the area of the entry hole in the bone. In the case of inlet damage to the anterior and posterior walls, the RA coefficient for H&N Baracuda Hunter pellets is the highest, and for conical-shaped pellets, it is the lowest (Fig 13).

### Gunshot damage to the lateral surface of the shaft of the porcine femur

Analyzing the shots fired into the lateral surface of the bone shaft, it should be noted that there is considerable variation in the thickness of the bone tissue of this wall. Ricochets after hitting the lateral side of the shaft occurred only in the case of shots fired with H&N Baracuda Hunter pellet (Table 7). In the case of entry wound damage to the bone, the pattern already described for shots to the anterior and posteriori surfaces of femur using 6.35 mm cal. airgun pellets is repeated. The damage with the largest surface area is produced by H&N Baracuda Hunter pellet, while the damage with the smallest surface area is, interestingly, produced by H&N Baracuda pellet (Fig 18). In case of periosteal entry damage, the biggest damage area was created with H&N Silverpoint and H&N Baracuda Hunter, the smallest by H&N Baracuda (Fig 19). A similar situation was observed for the RA coefficient (Fig 20). The largest values were observed for H&N Silverpoint and the smallest for H&N Baracuda. This is an interesting result, not being part of the trend seen in the case of shots of the anterior and posterior surfaces of the femoral shaft. Perhaps it is due to the design of the H&N Spitzkugel pellet, nevertheless this problem needs to be investigated in a separate experiment. The morphology of the gunshot damage is similar to that described in the case of a shot from the front or rear. Only the H&N Baracuda pellets pierced the bone through.

All the pellets, after hitting the shaft of the femur (regardless of the surface), caused damage to the periosteum. This damage was visualised in photographs of the dissected bone (Fig 21) as well as can be seen in photographs of the bone in ballistic gelatine. In all cases, they took on the appearance of a cloud of soft tissue, air bubbles and shrapnel fragments that surrounded the entrance hole.

It is also worth mentioning (although not related to the main topic of the paper) that the majority of lead pellets (mainly H&N Baracuda and H&N Baracuda Hunter, less frequently H&N Silverpoint), when hitting bone, fragmented, disintegrating into fragments of various sizes and shapes. This was not just a severed pellet cup, but also numerous small fragments, as seen, for example, in Fig 22 and Fig 23. Pellet fragments were found both in front of and to the side of the bone shaft, as well as inside the marrow cavity, as seen in Fig 24.

Analyzing the results obtained from gunshots of the anterior, posterior and lateral surfaces of the porcine femoral shaft, it can be seen that the morphology of the injuries is similar to gunshot damage inflicted by firearms (Long W.T. et al. [20]). Based on the available literature and research results, there is a gradual gradation of damage with increasing impact energy of airgun pellets, with pellets hitting with an energy of about 15.3J (Wightman G. et al. [4]) causing no visible damage to the long bone, and airgun pellets with impact energies of about 55J, respectively, depending on the type of airgun pellets, an increasing range of damage, which in the case of H&N Baracuda pellets resembled gunshot damage [17,19,20]. Tests conducted on bovine bone fragments using low-power pneumatic devices (Wightman G. et al [4]) are impossible to compare due to the different methodology and the researchers’ use of only a fragment of bovine femoral shaft embedded in ballistic gelatine, as well as the low energy of the airgun pellets, which did not cause detectable, morphologically noticeable damage to bone tissue. The results of the study are similar to those obtained in the work of Nguyen T-T. N. et al, [5], in which test shooting was performed on the shaft of a porcine femur embedded in ballistic gelatine using a so-called FSP (fragment simulating projectile) with mass of 0.78g, which, striking the bone at a velocity of approximately 325m/s caused perforation damage to the bone tissue, however, it did not enter the marrow cavity. The projectile was cylindrical in shape, thus different from the pellets used in our study. A single shot was made into an axially loaded porcine femur. The premise of this study, however, was not to assess the biological effects of an airgun shot, but injuries caused by metallic shrapnel with relatively high velocity but limited penetration ability, with an indication of explosive injuries caused by the detonation of explosives, unexploded ordnance or mines – i.e., issues relevant to military medicine [21–23]. The researchers did not describe the characteristics of the entry damage. This study, although similar in concept to the one we conducted, was performed on a single model, making it impossible to make a comparison with our results.

The adopted research model of the human thigh, using fresh pork femur embedded in ballistic gelatine, made it possible to determine the characteristics of gunshot damage inflicted by various types of airgun pellets of 6.35 mm calibre and significant impact energy. Variuos damage to the bone shaft was found in the form of multi-fracture fractures, longitudinal linear fractures, breakage of bone fragments – depending on the type of ammunition used, paralel to findings in previous studies [24]. Radiological results were similar to these reported in literature [25]. In addition, it was possible to assess the depth of penetration of the shot, which is related to the deforming and penetrating properties of individual types of pellet, as well as the behaviour of the pellets after hitting the bone in the context of their deformability, predisposition to ricochet and fragmentation of the projectile. Individual properties of selected pellets, representative for different groups of airgun pellets can be not only of cognitive importance in terms of ballistic properties, but also differentiation and identification valuable for forensic medicine [26]. The morphology of gunshot wounds inflicted from airguns using cal. 6.35 mm ammunition, resembles the characteristics of gunshot wounds from firearms, which can lead to misjudgments and misinterpretations. Therefore, the obtained results provide valuable cognitive material that can be used in forensics and forensic medicine when studying the effects of air weapons and differentiating from gunshot injuries from firearms.

The results obtained are an extension of our previous research [8]. Compared to earlier studies, we used different types of pellets (except for H&N Baracuda) with a larger calibre of 6.35 mm. The results obtained indicate a significantly greater range of damage generated by 6.35 mm calibre pellets due to their larger calibre and much higher impact energy (59 J vs. 44 J).

The experimental results obtained using a model of the human thigh confirm that air weapons pose a real danger to the health and life of humans and animals. The kinetic energy of the fired projectiles is sufficient to cause deep penetration in soft tissues and the associated real danger of suffering damage to internal organs with particular emphasis on vital ones. Given that the shaft of the femur, the strongest and largest bone in the mammalian body, is a tissue that is resistant to mechanical trauma, the results obtained in the experimental studies provide sufficient grounds for assuming that a gunshot from a 6.35 mm airgun can cause fatal injuries. Issues concerning the evaluation of the depth of tissue penetration with the use of a gelatine block will be the subject of further detailed research. The presented results are a valuable cognitive element in the field of ballistics and forensic traumatology. They may provide evidence that confirms the legitimacy of adopting a certain legal and criminal qualification of a criminal act in situations involving the use of air weapons. A separate finding is the dimension of public safety in a situation of increasing prevalence and relatively easy availability of pneumatic weapons, as well as pneumatic devices that can be modified to increase their kinetic energy beyond the permissible limit.

Our study has certain limitations. Firstly, we did not use human femurs in the study (mainly for legal reasons). As indicated in the aforementioned literature, human femoral shafts are slightly narrower than pig femoral shafts, but have much thicker cortical bone layers compared to pig femoral bones. This may contribute to the higher resistance of human femoral shafts to mechanical damage. Another limitation is the non-use of human thighs containing human femurs as a component in the study. Ballistic gelatine, even under ideal conditions, comes close to the properties of thigh muscles, but it lacks the structural complexity (nerves, blood, fascia) and elasticity/tearing properties of real tissue, often expanding cavities more and collapsing slower than flesh [27]. A separate problem is the small sample size of research groups, which limits the possibility of generalizability of the results

A strength of this study is that all three projectiles tested were of the same calibre, material and almost identical impact energy. Airgun pellets differed from each other in mass and nose design. It was also compared how different cortical thickness of the bone impact entrance wound damage extent.

The data shown in this paper can serve as a basis for further research, the purpose of which may be to develop a database of gunshot injuries with airgun pellets of various calibres and types. Such information can be useful to forensic physicians and the police in identifying the type of airgun pellet and weapon with which a person or animal was shot, finding application for medico-forensic and forensic findings.

## Conclusions

1. Gunshot damage to the to the bone and periosteum shaft of a porcine femur depends on the type of airgun pellets used to inflict the damage regardless of the bone shaft surface
2. The Baracuda Hunter pellets caused bone and periosteum damage of the gratest magnitude for the anterior and posterior surfaces of the femoral shaft.
3. Airgun pellets of penetrating type caused bone and periosteum damage of the least magnitude for the anterior and posterior surfaces of the femoral shaft.
4. In the case of the lateral surface of the shaft, significant variation in the thickness of the bone layer caused H&N Silverpoint airgun pellets to generate the greatest entry damage amond the pellets used in the study.

## Supporting information

S1 File Data. (XLSX)

## References

[1] Siemaszko A. Kogo biją, komu kradną Przestępczość nierejestrowana w Polsce i na świecie. Warsaw: Institute of Justice. 2001.

[2] Huelke DF, Harger JH, Buege LJ, Dingman HG. An experimental study in bio-ballistics: femoral fractures produced by projectiles--II. Shaft impacts. J Biomech. 1968;1(4):313–21. doi: 10.1016/0021-9290(68)90025-0 16329434

[3] DiMaio V. Gunshot Wounds: Practical Aspects of Firearms, Ballistics, and Forensic Techniques. 3rd ed. CRC Press. 2015.

[4] Wightman G, Beard J, Allison R. An investigation into the behaviour of air rifle pellets in ballistic gel and their interaction with bone. Forensic Sci Int. 2010;200(1–3):41–9. doi: 10.1016/j.forsciint.2010.03.025 20413234

[5] Nguyen T-TN, Tear GR, Masouros SD, Proud WG. Fragment penetrating injury to long bones. In: AIP Conference Proceedings, 2018. 090011. doi: 10.1063/1.5044868

[6] Nguyen T-TN, Carpanen D, Rankin IA, Ramasamy A, Breeze J, Proud WG, et al. Mapping the Risk of Fracture of the Tibia From Penetrating Fragments. Front Bioeng Biotechnol. 2020;8:544214. doi: 10.3389/fbioe.2020.544214 33042964 PMC7525181

[7] Tambuzzi S, Mazzarelli D, Gibelli D, Panigada G, Merelli V, Cattaneo C. Pellet gun trauma: An unusual and unexpected type of bone lesion. J Forensic Leg Med. 2022;88:102353. doi: 10.1016/j.jflm.2022.102353 35483249

[8] Wilk M, Chowaniec M, Chowaniec E, Bajor G. Analysis of gunshot damage to the porcine femur in a human thigh model using 5.5 mm airgun pellet: 3D reconstruction of gunshot injuries. PLoS One. 2025;20(7):e0328767. doi: 10.1371/journal.pone.0328767 40680074 PMC12273974

[9] Jussila J. Preparing ballistic gelatine--review and proposal for a standard method. Forensic Sci Int. 2004;141(2–3):91–8. doi: 10.1016/j.forsciint.2003.11.036 15062946

[10] cited online 14.12.2025. https://www.hn-sport.de/en/air-gun-hunting/baracuda-25

[11] cited online 14.12.2025. https://www.hn-sport.de/en/air-gun-hunting/baracuda-hunter-25

[12] cited online 14.12.2025. https://www.hn-sport.de/en/air-gun-hunting/silver-point-25

[13] cited online 14.12.2025. https://europeairguns.com/en/air-rifles-pcp/fx-bobcat

[14] cited online 14.12.2025. https://lmbr.pl/products.html

[15] Pillai TJ, lakshmi Devi CK, Devi TS. Osteometric Studies on Human Femurs. IOSRJDMS. 2014;13(2):34–9. doi: 10.9790/0853-13213439

[16] Hokari S, Mochizuki T, Tanifuji O. Three-dimensional cortical thickness in femoral diaphysis for ealthy elderly and osteoarthritic knees in women. Osteoarthritis and Cartilage. 2018;26:S459–60. doi: 10.1016/j.joca.2018.02.870

[17] Niimi R, Kono T, Nishihara A, Hasegawa M, Matsumine A, Kono T, et al. Cortical thickness of the femur and long-term bisphosphonate use. J Bone Miner Res. 2015;30(2):225–31. doi: 10.1002/jbmr.2345 25156261

[18] Ballistic gellatine mixing procedures practiced by the FBI. cited online 14.12.2025. https://press.hornady.com/release/2012/04/20/ballistic-gelatin-mixing-procedures-practiced-by-the-fbi/

[19] Koch CJ. The laws of bone architecture. American Journal of Anatomy. 1917;21:177–298.

[20] Long WT, Chang W, Brien EW. Grading system for gunshot injuries to the femoral diaphysis in civilians. Clin Orthop Relat Res. 2003;408:92–100. 10.1097/00003086-200303000-00010 12616044

[21] Riehl JT, Connolly K, Haidukewych G, Koval K. Fractures Due to Gunshot Wounds: Do Retained Bullet Fragments Affect Union?. Iowa Orthop J. 2015;35:55–61. 26361445 PMC4492146

[22] Bland-Sutton J. Observations on injuries of the bones of the limbs by the S. bullet. BMJ Military Health. 1915;24:314–23.

[23] Clasper J. The interaction of projectiles with tissues and the management of ballistic fractures. J R Army Med Corps. 2001;147(1):52–61. doi: 10.1136/jramc-147-01-05 11307677

[24] DiMaio V. Gunshot wounds: practical aspects of firearms, ballistics, and forensic techniques. 3rd ed. CRC Press. 2015.

[25] DiMaio V. Gunshot Wounds: Practical Aspects of Firearms, Ballistics, and Forensic Techniques. 3rd ed. CRC Press. 2015.

[26] Warlow TA. Firearms, the Law, and Forensic Ballistics. 3 ed. CRC Press. 2012.

[27] Jin Y, Mai R, Wu C, Han R, Li B. Comparison of ballistic impact effects between biological tissue and gelatin. J Mech Behav Biomed Mater. 2018;78:292–7. doi: 10.1016/j.jmbbm.2017.11.033 29195221

